# Mutation Detection with Next-Generation Resequencing through a Mediator Genome

**DOI:** 10.1371/journal.pone.0015628

**Published:** 2010-12-31

**Authors:** Omri Wurtzel, Mally Dori-Bachash, Shmuel Pietrokovski, Edouard Jurkevitch, Rotem Sorek

**Affiliations:** 1 Department of Molecular Genetics, Weizmann Institute of Science, Rehovot, Israel; 2 Department of Plant Pathology and Microbiology, Faculty of Agricultural, Food and Environmental Quality Sciences, The Hebrew University of Jerusalem, Rehovot, Israel; Tel Aviv University, Israel

## Abstract

The affordability of next generation sequencing (NGS) is transforming the field of mutation analysis in bacteria. The genetic basis for phenotype alteration can be identified directly by sequencing the entire genome of the mutant and comparing it to the wild-type (WT) genome, thus identifying acquired mutations. A major limitation for this approach is the need for an *a-priori* sequenced reference genome for the WT organism, as the short reads of most current NGS approaches usually prohibit *de-novo* genome assembly. To overcome this limitation we propose a general framework that utilizes the genome of relative organisms as mediators for comparing WT and mutant bacteria. Under this framework, both mutant and WT genomes are sequenced with NGS, and the short sequencing reads are mapped to the mediator genome. Variations between the mutant and the mediator that recur in the WT are ignored, thus pinpointing the differences between the mutant and the WT. To validate this approach we sequenced the genome of *Bdellovibrio bacteriovorus* 109J, an obligatory bacterial predator, and its prey-independent mutant, and compared both to the mediator species *Bdellovibrio bacteriovorus* HD100. Although the mutant and the mediator sequences differed in more than 28,000 nucleotide positions, our approach enabled pinpointing the single causative mutation. Experimental validation in 53 additional mutants further established the implicated gene. Our approach extends the applicability of NGS-based mutant analyses beyond the domain of available reference genomes.

## Introduction

Next-generation sequencing (NGS) technologies have revolutionized the field of microbial genomics and genetics [1]. It is now affordable to sequence an entire prokaryotic genome in order to identify acquired mutations [2]. For this, the millions of short reads produced by NGS are mapped to an *a-priori* sequenced reference genome of the wild-type (WT) [3] and mutations are inferred from the differences between the WT reference and the sequenced mutant [4].

Several studies have utilized NGS for identifying mutations. For example, isolates of the ethanol producing yeast *Pichia stipitis* were sequenced to detect mutations that facilitated efficient fermentation [5]. In another study, the geographical transmission of methicillin-resistant *Staphylococcus aureus* (MRSA) was traced, across a timescale of years, by genome-wide profiling of mutations in multiple isolates [6]. Evolution of bacterial symbionts [7] and pathogenic strains in the laboratory [8] were also studied by whole genome NGS.

A major barrier for identifying mutations through sequencing is the inherent dependence on a high-quality reference genome, and while, so far, over 1,200 genomes were fully sequenced, most isolated organisms lack a reference genome [9]. Since NGS data is characterized by short sequencing reads, usually 25-100 base pairs (bp), constructing *de novo* assemblies from the reads is not trivial [10], and as illustrated by Butler et al., assembling a full genome from unpaired short-sequencing-reads is often theoretically impossible [11].

Here, we present a general framework for identifying mutations using NGS without requiring an *a-priori* reference genome of the WT organism. This method utilizes a related genome, denoted the ‘mediator’, to which NGS data of both the WT and the mutant are mapped. We applied our method on the organism *Bdellovibrio bacteriovorus* 109J (henceforth 109J), for which no reference genome exists. *Bdellovibrio* is an aerobic δ-proteobacteria that presents an obligatory parasitic lifecycle, in which it feeds on gram-negative bacteria [12]. While WT *Bdellovibrio* is an obligatory predator, facultative host-independent mutants (HI) that can grow without the need for bacterial prey can be readily isolated in the laboratory [13]. A single gene implicated in the HI phenotype, the *hit* locus, has previously been characterized, and mutations in this gene were shown to be associated with the HI phenotype [12], [14]. We set out to characterize genomic alterations in HI mutants using the mediator-based re-sequencing approach.

## Results

### A mediator-based approach for mutations detection

**Figure 1:**
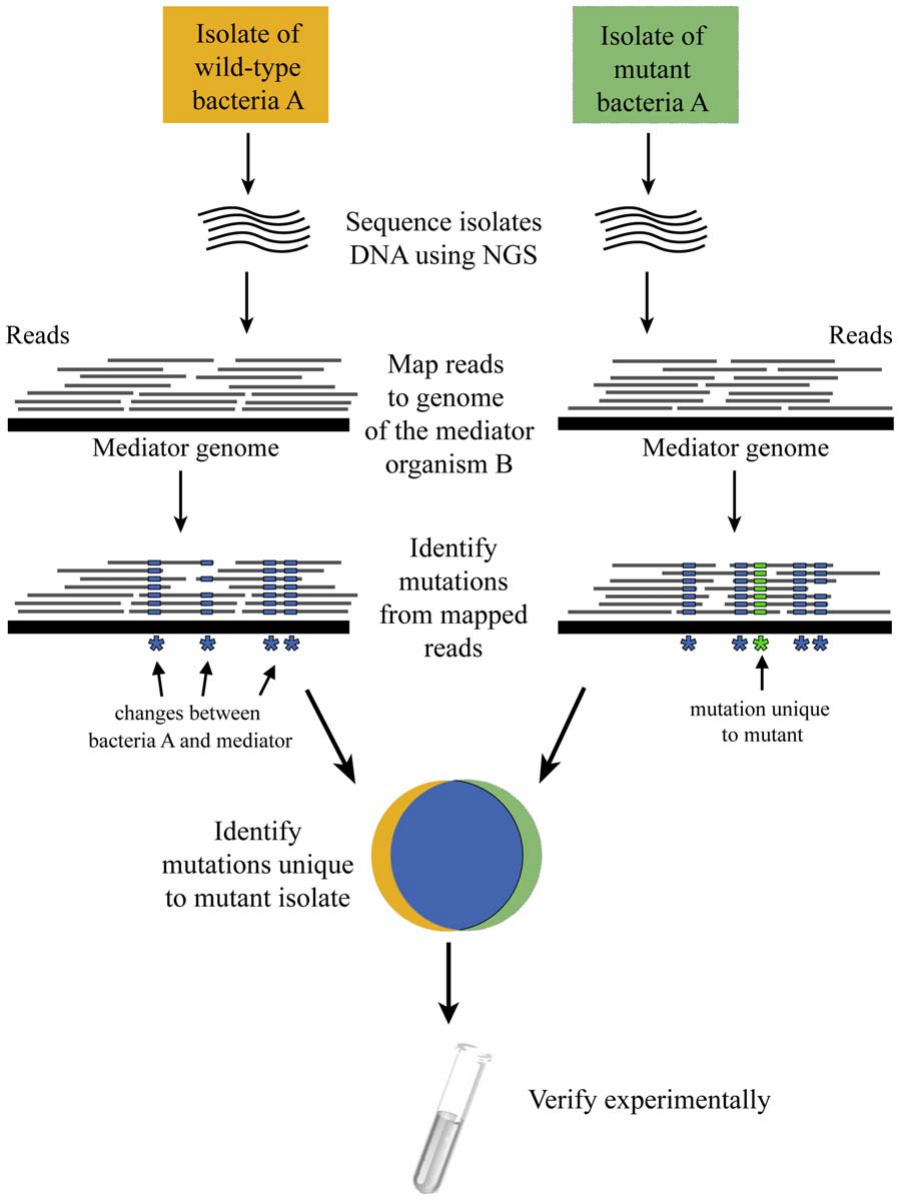
Workflow for mediator based resequencing. WT and mutant isolates are separately sequenced by NGS. The resulting short sequencing reads are projected onto the mediator genome. The mapped reads allow inferring variations between each of the isolates and the mediator genome (blue asterisks, mutations common to both the WT and mutant bacteria; green asterisk, mutation unique to mutant isolate). Mutations common to both isolates are discarded, leaving those that are unique to the mutant isolate. Finally, unique mutations to the mutant isolate are experimentally validated.

Our approach utilizes a mediator genome to pinpoint differences between mutant and WT isolates (Figure 1). Within this framework, genomic DNA of mutant and WT isolates are subject to whole-genome sequencing, and the resulting sequences of both isolates are separately mapped to the genome of the mediator organism. The high sequence coverage generated by NGS for bacterial genomes allows detection of local base differences without the need for whole genome assembly; positions that are consistently different between the mapped reads and the mediator genome are marked as genetic changes (Figure 1). This process resolves the differences between the genome of each of the sequenced isolates and the mediator genome, and produces a list of genomic differences both for the WT and the mutant strains. Genomic differences common to both isolates represent the evolutionary distance between the WT and the mediator and are therefore discarded, while changes unique to the mutant are further investigated, as they may be causative.

### Applying mediator-based sequencing to *Bdellovibrio* host-independent mutants

**Figure 2:**
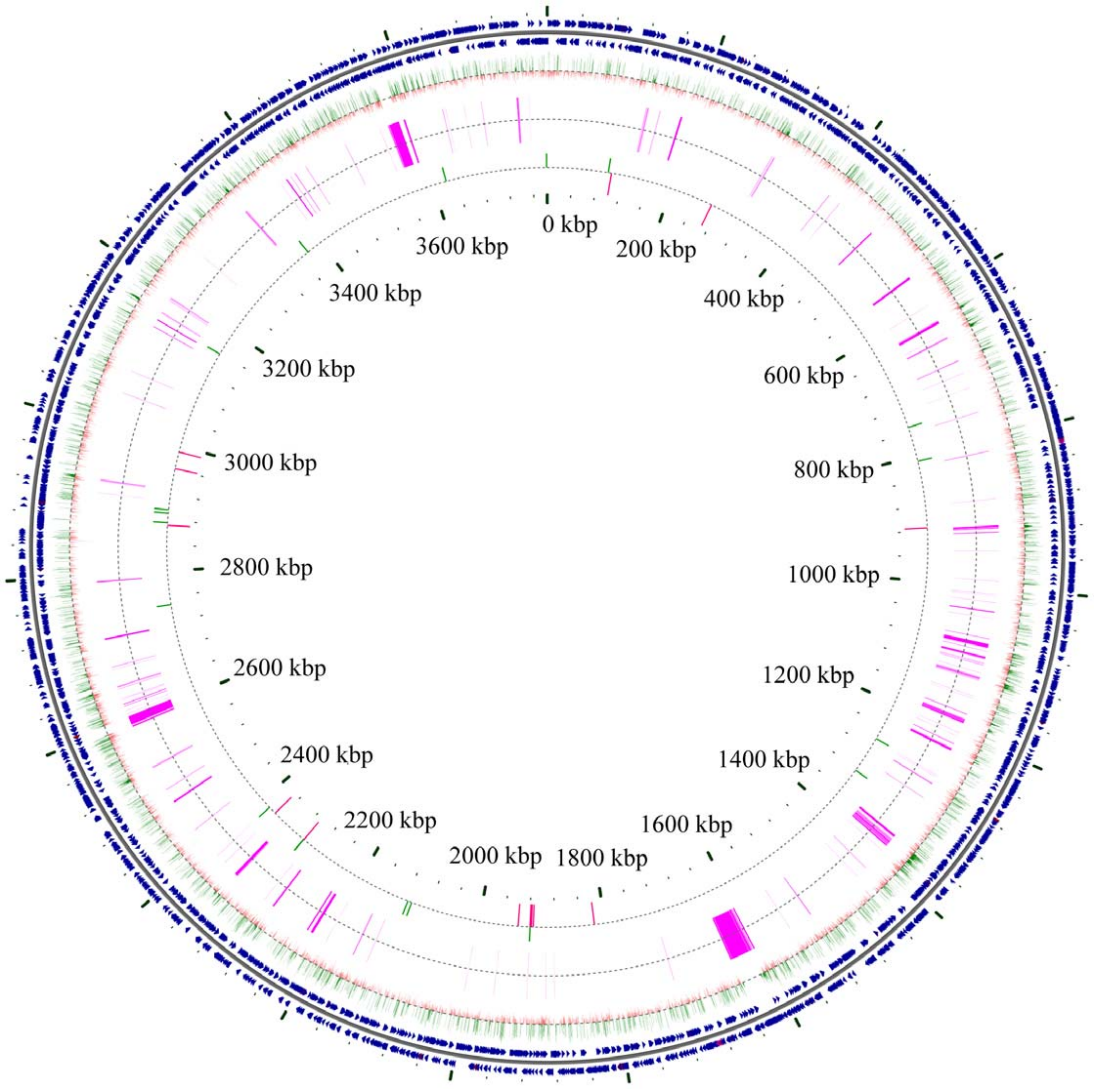
Genetic variation between *Bdellovibrio bacteriovorus* 109J and HD100. Shown is a genomic comparison between the sequenced strain 109J and the reference (mediator) HD100. Outer circles (blue) represent genes on the plus and minus strands of the 3,782,950 bp genome of HD100 (NC_005363). Middle circles represent the 28,379 genetic changes between WT 109J and WT HD100; green ticks, synonymous or intergenetic changes (85.7%); red ticks, non-synonymous changes (14.3%). Large deletions common to the WT 109J and HI 109J are in purple. The inner-most circle represents mutations unique to the HI or WT 109J clones; variations unique to HI are red, and those unique to the WT are green. (figure produced using CGView [15]).

To identify the mutation(s) that led to the host-independent (HI) phenotype in *Bdellovibrio bacteriovorus* 109J, we sequenced a single HI isolate using the Illumina Genome Analyzer platform. The sequencing produced >12 million reads of 33 bp, which could not be assembled into a reasonable number of contigs using widely used *de-novo* assemblers (Methods S1; Table S1; Figure S1). Since no reference genome is available for the 109J, the reads were mapped to the ‘mediator’ genome of *Bdellovibrio bacteriovorus* HD100 (HD100), the only available genome of the *Bdellovibrio* genus. Over one third of the reads were aligned to the mediator genome with 1 or more mismatches (Methods), reflecting the phylogenetic distance between the sequenced HI mutant and the mediator. Indeed, we were able to identify 28,386 single nucleotide differences between the HD100 genome and the sequenced HI mutant (Methods, Figure 2), with most of the detected changes (80.2%) being synonymous. In addition, 150,223 bases (4% of the genome) were not covered by any sequencing read, possibly reflecting DNA fragments that were deleted from the 109J isolate, or that became too variable to allow mapping of the 33 bp reads.

Obviously, this large amount of genetic variation does not allow identification of the specific causative mutations that lead to the phenotypic change. We therefore used the Illumina Genome Analyzer to sequence an isolate of WT 109J and mapped the resulting reads to the same mediator genome (Methods, Table S1, Figure S2). This revealed 28,379 single nucleotide differences between the mediator and the 109J WT, very close to the number identified in the HI mutant. Of these mutations, 28,367 (99.9%) were identical between the WT and the mutant, and thus most probably reflect the evolutionary changes since the separation of the mediator (HD100) and the WT (109J) strains. Discarding the common mutations revealed only 19 mutations unique to the HI mutant, a number small enough for downstream experimental validation (Table 1). This process also identified 12 mutations unique to the WT (Table S2). No large deletion was found to be unique to the HI mutant (Methods), pointing to one or more of the 19 identified single base changes and/or small insertion as the possibly causative mutations.

**Table 1:** Summary of detected mutations unique to the HI isolate.

Position on HD100	Description of mutated gene	Locus	Codon^a^		Amino acid	
			WT	Mutant	WT	Mutant
96981	hit locus ORF4	*Bd0108*	2 bp insertion, frame shift and a substitution from A to C (figure 3).			
752169	DNA polymerase I	*Bd0802*	ATC	ATt	I	I
807701	alkylphosphonate ABC transporter	*Bd0859*	GGG	tGG	G	W
1262908	histidine kinase	*Bd1335*	GGG	aGG	G	R
1322643	nucleic acid binding protein	*Bd1395*	CGC	tGC	R	C
1918689	Intergenic	N/A	A	G	N/A	N/A
2110823	hypothetical protein	*Bd2212*	TCT	TtT	S	F
2117292	Na/Pi-cotransporter family protein	*Bd2221*	GGA	Gag	G	E
2309751	hypothetical protein	*Bd2403*	GGG	GaG	G	E
2309974	hypothetical protein		ACG	cCG	T	P
2383979	Intergenic	N/A	G	A	N/A	N/A
2746436	two-component hybrid sensor	*Bd2838*	TAT	TgT	Y	C
2876741	30S ribosomal protein S12	*Bd2981*	AAG	AgG	K	R
2891582	elongation factor Tu	*Bd2994*	CAT	CAa	H	Q
2897108	Intergenic	N/A	C	A	N/A	N/A
3159141	Intergenic	N/A	C	T	N/A	N/A
3373629	poly(A) polymerase	*Bd3464*	GAC	aAC	D	N
3621096	ATP-dependent protease LA	*Bd3749*	ATG	ATt	M	I
3781899	60 KD inner-membrane protein	*Bd3912*	TCC	TaC	S	Y

a lower case letter represents mutated base.

### Experimental validation of identified mutations

**Figure 3:**
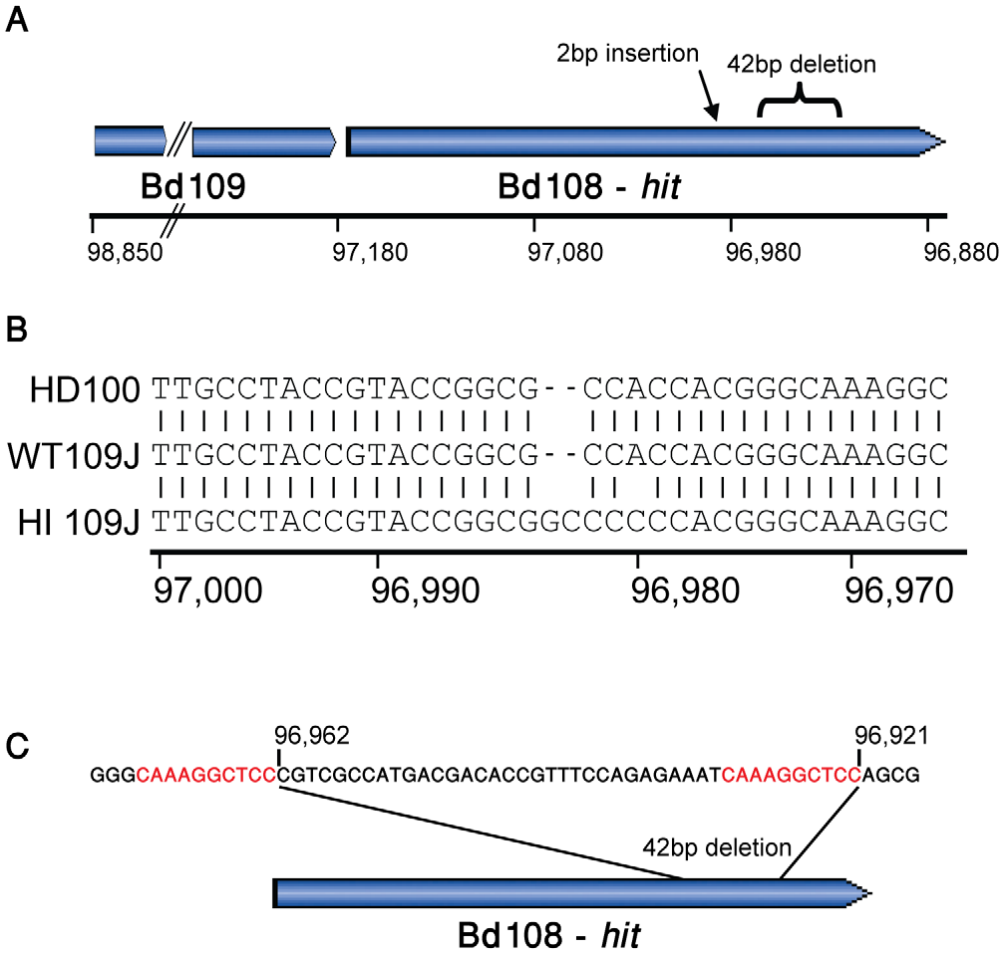
Mutations at the *hit* locus in 54 host independent mutants. (**A**) The genomic vicinity of the *hit* locus (locus tag Bd108). X-axis, coordinates relative to the reference HD100 strain (Genbank accession: NC_005363) (**B**) A 2 bp insertion is observed in the Illumina-sequenced host independent (HI) mutant but not in the WT. (**C**) A 42 bp deletion at the *hit* is observed in 46% of sequenced HI mutants. Shown is a 10 bp direct repeat flanking the deletion, which presumably mediates recombination-based deletion.

All non-synonymous mutations in the HI stain were tested and verified using directed PCR followed by Sanger sequencing. Interestingly, one of the detected mutations, a 2 bp insertion leading to a frameshift, occurred at the *hit* locus that was previously implicated as essential for host dependence in *Bdellovibrio* [14] (Figure 3). This insertion in the hit locus was therefore suspected as the mutation associated with the HI phenotype, based on the estimates that HI phenotype appearance is usually the result of a single mutational event [14].

To further assess whether a mutation at the *hit* locus is indeed associated with host independent phenotype, we sequenced the entire *hit* locus in 53 additional HI isolates. Most of these clones (89%) were indeed found to harbor point mutations, small deletions and insertions leading to frameshifts, or larger deletions, at the *hit* locus (Table 2). Five of these mutations were identical to the 2 bp insertion we identified in the first HI clone (Figure 3B). These data imply that in the majority of cases the HI phenotype is caused by a mutation at the *hit* locus; however, mutations occurring in other loci at lower frequencies (11% of the cases) may also result in host independence.

**Table 2:** Composition of *hit* locus ORF4 in 54 HI isolates.

Sequence of the *hit* locus	Number of clones (percent of total)
Frame shift	19 (35%)
Deletion of 42 bp	25 (46%)
Stop codon	3 (6%)
Point mutation	1 (2%)
No mutation in *hit*	6 (11%)
**Total clones**	**54**

Interestingly, the most common mutation in *hit* was a 42 bp deletion, occurring in 46% of the HI isolates (Figure 3C; Table 2). We found that this deletion is flanked from both sides by a 10 bp direct repeat. Possibly, repeat-mediated recombination [16] is the driver of this deletion, and this might explain the exceptionally high frequency (10^−2^, [17]) of host-independence appearance.

To further examine whether one of more of the 18 remaining genomic changes observed in the HI mutant (except for the *hit* locus) are linked to the HI phenotype, we randomly selected one of the 6 isolates in which no *hit* mutation was found. In this new HI mutant we sequenced the full genes that were mutated in the original HI (or their surrounding regions, when they were placed in intergenic regions). None of these 18 mutations were detected in the new mutant, suggesting that these are not linked to the HI phenotype. These results further pinpoint the mutation we detected at the *hit* locus as the single mutation associated with the HI phenotype in the HI mutant sequenced by NGS.

## Discussion

NGS provides powerful, unbiased means for identifying all mutations in an organism in a single sequencing run, but usually requires an *a-priori* sequenced reference genome to allow template-mediated assembly. Here, we presented a method that extends the usage of NGS for organisms lacking a reference genome, by comparing the genomes of a WT isolate and a mutant isolate through a mediator organism. In the case of *B. bacteriovorus*, this strategy allowed pinpointing a minimal set of candidate causative mutations out of over 28,000 genetic differences between the tested organism (strain 109J) and its closest sequenced relative (strain HD100).

While our approach can reliably identify point-mutations, deletions, and small insertions, it is limited in its ability to identify large insertions. Sequencing reads that originate from insertions of genomic elements are unique to the sequenced clone, and will therefore not align to the mediator genome. This limits the comparison between the WT and the mutant to regions that exist also in the mediator genome. However, the maximal size of inserted sequences can be estimated by summing the lengths of the unaligned reads and dividing by the average coverage. In the case of *Bdellovibrio*, less than 10% of the reads did not align, indicating that our method is likely to identify at least 90% of the mutations in the109J HI genome.

Recent studies reported on successful *de-novo* assembly of bacterial genomes from NGS data, without the need for reference genomes. However, these mostly utilized paired-end sequencing, a combination of sequencing technologies, and very high sequence coverage, to achieve proper assembly. While such approaches do not necessitate reference or mediator genomes, at the current levels of sequencing costs they are less affordable for routine use in individual laboratories [10], [12].

While resequencing studies usually utilize a specialized short reads aligner (e.g. MAQ [18], SOAP [19], or ELAND (Illumina, unpublished), in this study we used BLAST [20] to align the short reads to the mediator reference genome. Although BLAST is much more computationally demanding, it provides a higher alignment flexibility that is crucial when the reference genome is expected to contain many differences as compared to the sequenced genome. Indeed, to reduce computational complexity and increase performance speed, specialized aligners usually do not map reads with more than 2–3 mismatches, and in some cases lack the ability to detect insertions and deletions [2]. Since in mediator-based resequencing a significant amount of mismatches is anticipated, BLAST seems to be a preferable alignment tool, as it poses almost no limitation on the number of mismatches (except for a required seed of at least 4 bp), and also allows detection of single-base insertions and deletions. We note, however, that in the absence of a significant computational power, BLAST is currently impractical for mediator-based resequencing of non-microbial genomes.

Whole genome sequencing via NGS is becoming a standard method for deciphering the genetic basis for phenotypic alternations in bacteria. Our mediator-based approach expands the spectrum of this method as a general, affordable solution for many prokaryotic species for which no direct reference genome is available. With future reduction of sequencing costs, this approach could ultimately also be used with eukaryotic genomes of larger sizes.

## Materials and Methods

### Bacterial strains and culture conditions

WT 109J was grown at 30°C in HEPES buffer (25 mM HEPES, 2 mM CaCl_2_.2H_2_O, 3 mM MgCl_2_.6H_2_O, pH 7.8) in two-member cultures with *Escherichia coli* ML35 as a prey. The host-independent (HI) isolate used for high-throughput sequencing is a spontaneous mutant able to grow axenically in a rich medium such as PYE (1% Bacto peptone, 0.3% yeast extract, 2 mM CaCl_2_.2H_2_O and 3 mM MgCl_2_.6H_2_O, pH 7.6) [13]. Fresh attack phase (AP) cells from the WT strain were obtained from overnight (O.N.) cultures by inoculating 100 ml HEPES buffer with 2.10^9^ colony forming units/ml of *E. coli* ML35 prey and 10^7^ predatory cells. WT AP cells were filtrated twice through a 0.45 µm filter (Sartorius) for separation from residual prey and debris. This procedure ensured that there were no residual *E. coli* cells contaminating our samples, as confirmed by phase contrast microscopy. HI was grown in PYE medium O.N. at 30°C.

Additional HI isolates were obtained as follows: i) using a traditional protocol in which fresh AP cells are filtered four times through a 0.45 µm filter (Sartorius), plated on PYE plates and incubated at 30°C until colonies appear (34 isolates), and; ii) using a recently described procedure in which *E. coli* diaminopimelic acid (DAP) auxotrophs are used as hosts. In this procedure, fresh AP cells are grown on PYE plates without DAP, and therefore neither the auxotroph *E. coli* nor the WT *Bdellovibrio* are able to grow on these plates, thus omitting the filtration step (19 isolates) [17]. The HI colonies isolated using the second procedure, were kindly provided by Daniel E. Kadouri (University of Medicine and Dentistry of New Jersey, NJ, USA).

### Genomic DNA preparation

Genomic DNA of WT and HI isolates were prepared using Wizard genomic DNA purification kit (Promega). The samples were prepared according to the manufacturer's protocol and submitted to the High Throughput Sequencing Unit in the Weizmann Institute of Science for sequencing with Illumina's Genome analyzer.

### Mapping of sequencing reads and identification of mutations

The reference genome of *Bdellovibrio bacteriovorus* HD100 (NC_005363) was downloaded from NCBI and used for mapping the reads produced from the 109J strain Illumina Genome Analyzer runs. The sequencing reads for each run were mapped separately using Blast (Blastall v2.2.20) with the following parameters [-p blastn -e 0.0001 -b 20 -v 20 -m 0 –W 11 -F F], allowing up to 6 alignment errors, and minimal alignment lengths of 27 and 30 bp for the 33 and 38 bp long sequencing reads, respectively. The shorter read length (33 bp) in the case of the HI mutant was compensated for by sequencing two Illumina lanes instead of one.

Detection of mutations was done by analyzing the alignments, and genomic positions which were consistently (>60%, coverage of 5 or more reads) different were marked as mutated. The relatively high coverage (x51 - x86) allowed testing the reproducibility of the alignment and thereby excluding alignment errors and sequencing errors over real changes. A mismatch table of the 109J strain against the HD100 strain was compiled separately for the HI and the WT samples, and was compared to identify unique mutations.

Due to the shorter length of reads produced for the HI clone, some regions, which exhibited high genetic variation, had little or no alignments. The lower coverage at hyper-mutated regions led to an initial identification of 23,502 mutations, with larger predicted deletions. We computationally scanned the coverage in the WT 109J data to identify hyper-mutated regions, which limited the alignment of the short sequencing reads obtained for the HI clone. These regions were inferred by locating extreme changes in coverage (defined as one standard deviation below the average) combined with clusters of mutations concentrated in small regions. Following identification of these regions we repeated the process of genetic variation discovery with relaxed thresholds for these specific regions.

## Supporting Information

Figure S1 **Distribution of contig lengths produced by *de novo* Velvet assembly of short sequencing reads.** Most contigs generated in the assembly stage were less than 2,000 bp long, with very few contigs spanning over 5,000 bp. The high number of generated contigs, obtained by running Velvet 0.7.55 on our data, renders this approach impractical for identifying genetic variation between the two almost identical clones.
(TIF)

Figure S2 **Distribution of read coverage mapped to the *Bdellovibrio bacteriovorus* HD100 genome.** Coverage of the genome largely deviates from the theoretical Poisson model. The vast majority of the mediator genome was covered by multiple reads, with only 4.12% of the genome not covered. Uncovered regions may represent large deletions in the 109J or regions that differ extremely between the sequenced and the mediator genomes. The less uniform coverage in the HI stems from the shorter read length.
(TIF)

Table S1 Summary of sequencing results.
(DOC)

Methods S1 ***De novo* assembly of *Bdellovibrio bacteriovorus* 109J sequencing data.**
(DOC)
