# Supplementary figures and images for: Mutation Detection with Next-Generation Resequencing through a Mediator Genome
Source: PLoS One. 2010 Dec 31;5(12):e15628. doi: 10.1371/journal.pone.0015628 (PMC3013116; doi:10.1371/journal.pone.0015628)

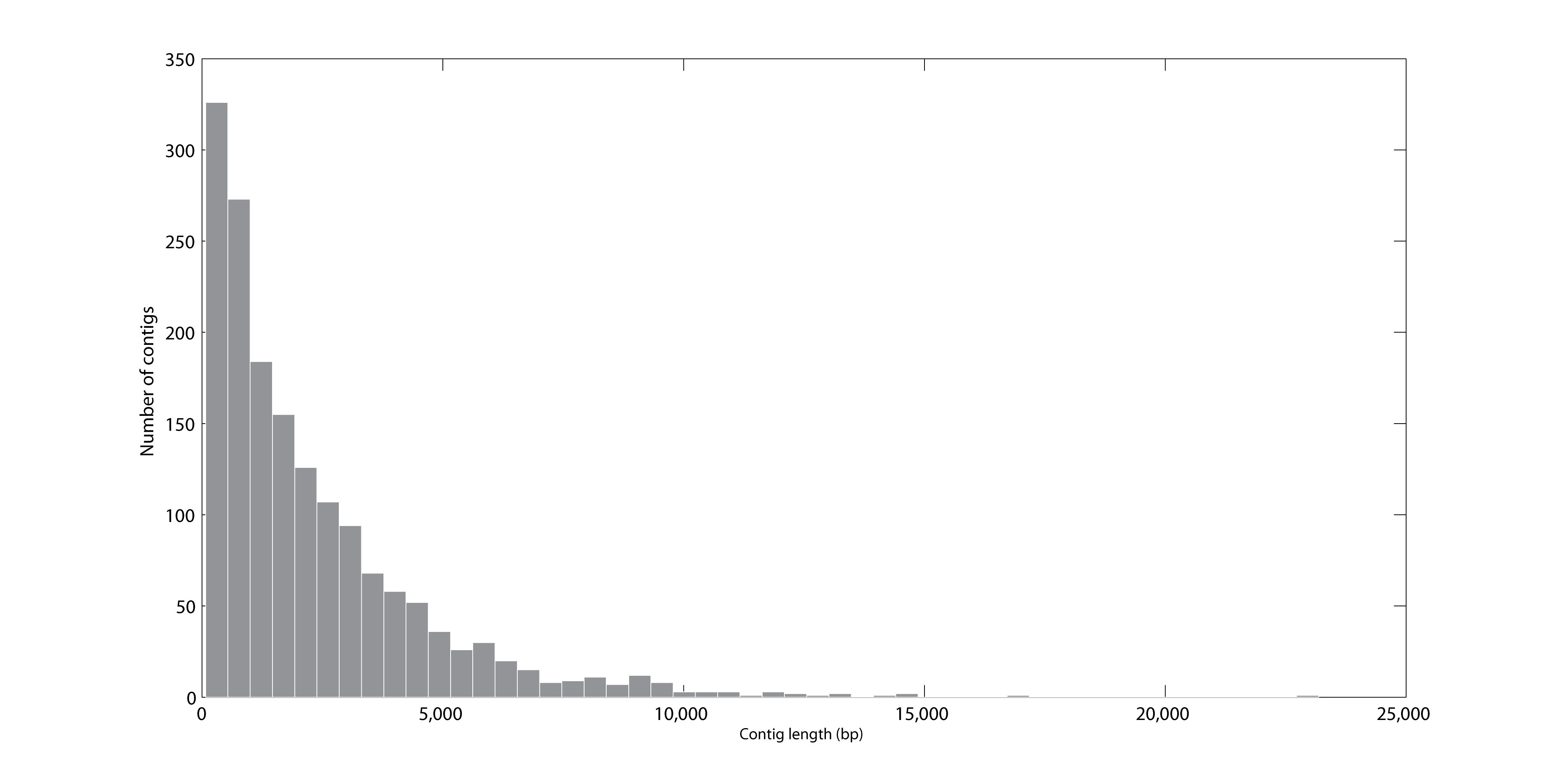

Supplement: Figure S1 — Distribution of contig lengths produced by de novo Velvet assembly of short sequencing reads. Most contigs generated in the assembly stage were less than 2,000 bp long, with very few contigs spanning over 5,000 bp. The high number of generated contigs, obtained by running Velvet 0.7.55 on our data, renders this approach impractical for identifying genetic variation between the two almost identical clones. (TIF) [file pone.0015628.s001.tif]

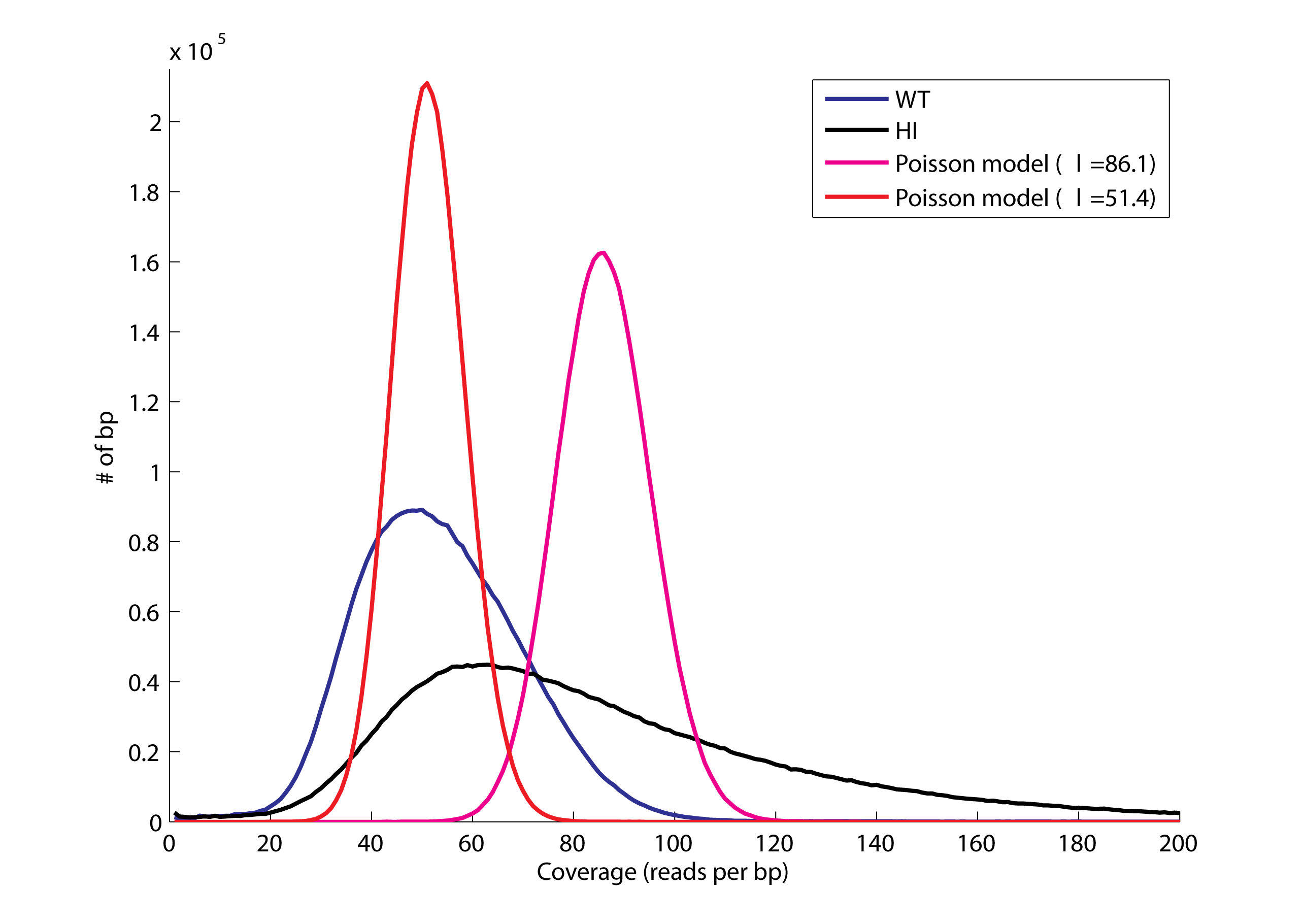

Supplement: Figure S2 — Distribution of read coverage mapped to the Bdellovibrio bacteriovorus HD100 genome. Coverage of the genome largely deviates from the theoretical Poisson model. The vast majority of the mediator genome was covered by multiple reads, with only 4.12% of the genome not covered. Uncovered regions may represent large deletions in the 109J or regions that differ extremely between the sequenced and the mediator genomes. The less uniform coverage in the HI stems from the shorter read length. (TIF) [file pone.0015628.s002.tif]
