# Supplementary material for: Mutation Detection with Next-Generation Resequencing through a Mediator Genome
Source: PLoS One. 2010 Dec 31;5(12):e15628. doi: 10.1371/journal.pone.0015628 (PMC3013116; doi:10.1371/journal.pone.0015628)
Supplement: Table S1 — Summary of sequencing results. (DOC) [file pone.0015628.s003.doc]

**Table S1. Summary of sequencing results**

| **Strain** | **Read**  **length** | **# of reads** | **# reads aligned** | **% reads aligned** | **Average**  **coverage** |
| --- | --- | --- | --- | --- | --- |
| **HI** | 33 bp | 12,289,594 | 10,039,201 | 81.7 | x86 |
| **WT** | 38 bp | 5,999,157 | 5,122,709 | 90.7 | x51 |
