# Supplementary material for: Mutation Detection with Next-Generation Resequencing through a Mediator Genome
Source: PLoS One. 2010 Dec 31;5(12):e15628. doi: 10.1371/journal.pone.0015628 (PMC3013116; doi:10.1371/journal.pone.0015628)
Supplement: Methods S1 — De novo assembly of Bdellovibrio bacteriovorus 109J sequencing data. (DOC) [file pone.0015628.s004.doc]

**Supplementary material**

***De novo* assembly of *Bdellovibrio bacteriovorus* 109J sequencing data**

To assess the ability of state-of-the-art assemblers to produce long contigs by non-experts, we tested one of the most widely used assemblers, Velvet, on our sequencing data . The latest version of Velvet was downloaded from the author's site (0.7.55, <http://www.ebi.ac.uk/~zerbino/velvet/>) and ran according to the instructions provided by the authors with different parameters according to the documentation (hash lengths of 15, 21, 25, and 29; minimal contig length 1 and 100; expected coverage 'auto' or 56, according to the preliminary alignment to the HD100 genome). The best assembly in terms of median contig length and total coverage created 1630 contigs with median length of 1662 bp, with the longest contig spanning 23,211 bp (Figure S1). The sum of the contig lengths was 3,855,253 bp, very close to the genome size of the fully sequenced *Bdellovibrio bacteriovorus* HD100 (3,782,950 bp). However, the high number of contigs makes this assembly hard to use, and without additional mate-pair data, it could not be readily optimized. Similar assessment with another widely used assembler, SOAPdenovo, resulted in an inferior assembly and results are therefore not presented here.

1. Zerbino DR, Birney E (2008) Velvet: algorithms for de novo short read assembly using de Bruijn graphs. Genome Res 18: 821-829.
